# The Use of the File in Dentistry

**Published:** 1867-01

**Authors:** W. H. Atkinson


### THE USE OF THE FILE IN DENTISTRY.

Read before the Society of Dental Surgeons of New York, December 19, 1866.

BY W. H. ATKINSON.

AUTOSCHEDIASTICAL as have been the efforts at original instruction in our specialty, yet we owe more to these "off-hand efforts" of earnest minds than to all the labored and belabored scholastic and pseudo scientific works of the mere copyists of the crudities in the principles and practice of the pioneers in our specialty. Many good things have been laid aside and many wretched ones sedulously preserved only to occupy space that should be left empty or filled with better things. The misuse of a good instrument may bring it into disrepute. This has been the case with the Dental File to an extent exceeding that of any other instrument or appliance ever used in Dentistry.

In certain classes of teeth the file, in judicious hands and faithfully used, is capable of saving them without any other instrument except stones and polishers to smooth the cut surfaces down to a fine finish, so that the brush and tooth pick can keep them so through life, there is scarcely an example of a Dentist who uniformly uses the file *sufficiently* in preparing and finishing cavities before and after filling.

No *corners* should be left by the file, but regularly rounded margins like the corners of the uninjured tooth should be preserved on all surfaces worked upon.

Filing cannot be safely performed with a coarse file and slow motion; fine cut shallow teeth, and quick, light, sprightly movements will leave a surface clean, and capable of taking a fine polish with stones and powders adapted to the purpose.

The file should always be kept wet during the operation, so as to wash off the debris as it falls from the mass from which it is taken, and distribute the heat produced by the arrested motion. Common tape, sand paper, and wash leather charged with some finishing powder, such as the flour of emery, corundum, tripoli, &c., &c., are indispensable means by which to trim down and polish the rounded and irregular surfaces of teeth and fillings, so as to insure facility of keeping them clean and nicely polished. Any filling that will not bear filing is unsafe and ought not be allowed to remain in a tooth. I am aware that this rule condems the vast majority of fillings as now inserted by those denominated " first class" —" none better," &c., &c.

I am also aware that it is not only possible, but practicable, to so nicely prepare and fill cavities that very little filing will be necessary. In fact, the very highest skill is able to dispense with the file as a finishing instrument, resting satisfied with Stones polishing powders, tapes and sticks. But it is questionable whether wasting a little more gold by having a little excess will not be a means of a strict economy of time, which is gold, and add to certainty and completeness of success in the end. It is not pleasant to the patient nor flattering to the ability and faithfulness of the operator, to be under the necessity of patching deficient portions of a filling, that were deemed perfect until the attempt to finish them revealed the soft spots and imperfections that conscience can not pass over when so *fully uncovered* to the operator *and* patient.

An affectionate regard for the truth and a persistent determination to come up to her requirements in our own practice as fast as they become known, will soon enable us to speak out our convictions so that they may be put to the test and our views confirmed or displaced by others more in accordance with the efficiency we are all so earnestly seeking.

The more I reflect upon the difference of apprehension existing among men, the more am I convinced that it consists in the difference of degree of intelligence they possess. Truth is a unit, when we arrive at the demonstration thereof, in the completeness of mental labor to the degree of knowing it to be truth, and then all with one accord pay it the homage which is its due.

In consequence of the unfortunate use made of the file, there are many Dentists and more people who are unwilling to even listen to its advocacy with the admission that it were possible to do other than harm to the teeth in its use; and, of course, truth to them demands that it be avoided in toto. While those who have been in the habit of filing, and having their teeth filed down to unsightly pegs, which they have retained ever since in vigorous use, are, of course, very strong partizans of the file; and the truth as they see it urges them very positively in the line of heroic filing.

These and those are but half truth, and of course not complete until brought together by an intelligent fusion, when lo the veritable truth stands forth revealed to and acknowledged by both. It will no more do to half file and half finish and then look for perfect results, than to half survey the field of facts upon which the demonstration of truth depends and then call the deduction the truth.

Perfect preparation for and faithful execution of professional work can alone entitle us to assume the responsibilities of a professional career.

When we *know* what is required of us, we are too honest to hesitate to take the necessary steps involved to fit us for a *clean* and honorable practice.

